# P-1054. Culturing air for viable fungi at hospitals within a healthcare system

**DOI:** 10.1093/ofid/ofaf695.1249

**Published:** 2026-01-11

**Authors:** Eileen Driscoll, Zachary Wilkins, Yoon Cho, Binghua Hao, Shaoji Cheng, Alexander Sundermann, Ashely Ayres, Graham M Snyder, M Hong Nguyen, Cornelius J Clancy

**Affiliations:** University of Pittsburgh, Pittsburgh, Pennsylvania; University of Pittsburgh, Pittsburgh, Pennsylvania; University of Pittsburgh, Pittsburgh, Pennsylvania; University of Pittsburgh, Pittsburgh, Pennsylvania; University of Pittsburgh, Pittsburgh, Pennsylvania; University of Pittsburgh, Pittsburgh, Pennsylvania; University of Pittsburgh, Pittsburgh, Pennsylvania; University of Pittsburgh, Pittsburgh, Pennsylvania; University of Pittsburgh Medical Center, Pittsburgh, Pennsylvania; University of Pittsburgh, Pittsburgh, Pennsylvania

## Abstract

**Background:**

Airborne fungi are important nosocomial pathogens. There are limited long-term longitudinal data on populations of viable airborne fungi in hospitals.

**Table 1.**
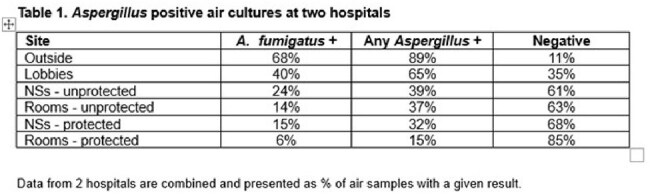
Aspergillus positive air cultures

**Table 2.**
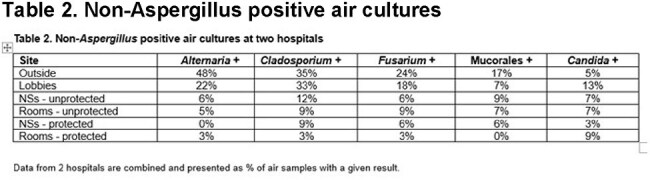
Non-Aspergillus positive air cultures

**Methods:**

We cultured air samples at least once/month from May 2023 through May 2025 at 2 hospitals separated by 1.5 miles using direct impact MYE agar plates and an SAS Super 100 (1,000 L). At both hospitals, we sampled the following sites: Outside, lobby, nurse stations (NS) and rooms on units housing immunosuppressed hosts. Rooms were protected (i.e., HEPA filter, positive pressure) and unprotected. Plates were incubated at 30°C, and fungi identified by morphology and ITS sequencing.

**Results:**

There was a clear hierarchy in air culture positivity for pathogenic moulds, which did not differ at the 2 hospitals: outdoor cultures > lobbies > NS > unprotected rooms > protected rooms. The predominant fungus was Aspergillus fumigatus (68% and 6% of outdoor and protected room samples positive, respectively), followed by non-fumigatus Aspergillus spp. Other common moulds were Alternaria (48% outside +), Cladosporium (35% outside +), Fusarium (24% outside +) and Mucorales (17% outside +, including Rhizopus, Syncephalastrum racemosum and Mucor). Candida was cultured at similar frequencies outside and inside hospitals ((overall + (range): 7% (3%-13%)); in rank order, spp. were C. glabrata (n=8), C. krusei (6), C. albicans (5), C. tropicalis (4), C. parapsilosis (3), C. auris (1). C. auris was recovered from a unit that was not known to house an infected pt.

**Conclusion:**

Potentially pathogenic fungi were commonly cultured from air samples inside and outside 2 hospitals in the same city, including within rooms housing immunosuppressed pts. Burdens were lower within hospitals than immediately outside. Fungi included moulds like Aspergillus spp., which are well-recognized airborne pathogens, and Candida spp., which are not typically considered as such. Among the latter was C. auris, which was temporo-spatially distant from pts known to be infected at the hospitals. We are currently studying potential links between fungi recovered during surveillance and invasive fungal infections, including by whole genome sequencing.

**Disclosures:**

M Hong Nguyen, MD, Basilea: Advisor/Consultant|BioMerieux: Grant/Research Support|Melinta: Grant/Research Support|Pulmocide: Advisor/Consultant|Pulmocide: Grant/Research Support Cornelius J. Clancy, MD, Merck: Grant/Research Support|Shionogi: Advisor/Consultant

